# Barriers to access and utilisation of HIV/STIs prevention and care services among trans-women sex workers in the greater Kampala metropolitan area, Uganda

**DOI:** 10.1186/s12879-020-05649-5

**Published:** 2020-12-07

**Authors:** Tonny Ssekamatte, John Bosco Isunju, Muyanga Naume, Esther Buregyeya, Richard K. Mugambe, Rhoda K. Wanyenze, Justine N. Bukenya

**Affiliations:** 1 Department of Disease Control and Environmental Health, Makerere University School of Public Health, Kampala, Uganda; 2 Programs Department, Transgender Equality Uganda, Kampala, Uganda; 3 Department of Community Health and Behavioural Sciences, Makerere University School of Public Health, Kampala, Uganda

**Keywords:** HIV/AIDS, Sexually transmitted infections, Trans-women, Sex work, Uganda

## Abstract

**Background:**

Trans-women sex workers bear the greatest brunt of HIV and other sexually transmitted infections (STI). Trans-women are 49 times more at risk of HIV infections compared to the general population. However, they remain underserved and continue to grapple with access to and utilisation of HIV/STI prevention services. This study explored barriers to access and utilisation of HIV/STI prevention services and associated coping mechanisms.

**Methods:**

This exploratory qualitative study was conducted among trans-women sex workers in the Greater Kampala Metropolitan area, Uganda. A total of 22 in-depth interviews, 6 key informant interviews and 9 focus group discussions were conducted to obtain data on barriers to access and utilisation of HIV and other STI prevention and care services, and coping strategies of trans-women sex workers. Data were analysed through thematic analysis using a hybrid of inductive and deductive approaches.

**Results:**

Individual level barriers to access and utilisation of HIV/STI prevention and care services included internalised stigma and low socio-economic status. Healthcare system barriers included social exclusion and lack of recognition by other key population groups; stigmatisation by some healthcare providers; breach of confidentiality by some healthcare providers; limited hours of operation of some key population-friendly healthcare facilities; discrimination by straight patients and healthcare providers; stockout of STI drugs; inadequate access to well-equipped treatment centres and high cost of drugs. At community level, transphobia hindered access and utilisation of HIV/STI prevention and care services. The coping strategies included use of substitutes such as lotions, avocado or yoghurt to cope with a lack of lubricants. Herbs were used as substitutes for STI drugs, while psychoactive substances were used to cope with stigma and discrimination, and changing the dress code to hide their preferred gender identity.

**Conclusions:**

Individual, community and healthcare system barriers hindered access and utilisation of HIV/STI prevention and care services among the trans-women sex workers. There is a need to create an enabling environment in order to enhance access to and utilisation of HIV/STI prevention and care services for trans-women sex workers through sensitisation of healthcare providers, other key population groups and the community at large on the transgender identity.

## Background

The trans-women, defined as individuals who were assigned the male sex at birth but identify as women [1], globally are at an elevated risk of sexually transmitted infections (STIs), including human immunodeficiency virus (HIV). More than 19.1% of the trans-women are living with HIV. In addition, the risk of HIV infection is 49 times higher among the trans-women compared to the general population [2]. In Sub-Saharan Africa, the HIV prevalence among the trans-women is as high as 59% [3]. Although there are no reliable national estimates for the HIV burden among the trans-women in Uganda, King, Nanteza [4] reported a prevalence as high as 20%.

Sex work, which is the main source of livelihood for majority of the trans-women [4, 5], is usually associated with high-risk behaviours such as incorrect and inconsistent condom use, having sex under influence of drugs and other substances [6, 7], and multiple sexual partnerships/relationships [8], which are all known to escalate the risk of HIV transmission [9, 10]. Thus, trans-women sex workers, who are the focus of this study, experience higher rates of STIs including HIV compared to non- trans-women sex workers [5, 11]. Besides, the high prevalence of STIs and HIV among the trans-women sex workers could be linked to the absence of a conducive environment for accessing prevention, care, and treatment services [12, 13]. The low education status, social exclusion [14] and lack of empowerment that are prevalent among the trans-women sex workers also make it difficult for them to negotiate for HIV prevention strategies such as the correct and consistent use of condoms [12, 15–17]. There is also evidence that social, cultural and policy issues such as internalized stigma and violence, limited access to non-stigmatizing healthcare services, inaccurate perceptions of self and partners’ risk escalate STI/HIV acquisition among the trans-women sex workers [14].

Whereas STIs and HIV are highly prevalent among the trans-women sex workers, there is limited evidence of the facilitators and barriers to access and utilization of STI/HIV prevention and care services globally. Majority of the key population studies focus on understanding access to HIV and prevention services among the men who have sex with men and female sex workers [18]. Besides, trans-women sex workers’ vulnerabilities, frustrations, and insecurities have been historically overlooked by mainstream society (Khan et al., 2009), including among healthcare providers [18]. For example, trans-women sex workers were (and are still in Uganda) originally misclassified as men who have sex with men (MSM), and remain absent from national HIV surveillance systems and program interventions [8].

In order to bridge the knowledge gap, our study used the social ecological model [19, 20] and the Andersen and Newman framework to understand the barriers to the utilisation of HIV/STI prevention and care services among trans-women sex workers in the greater Kampala metropolitan region. The social ecological model aids in describing the interactive characteristics of individuals and environments that underlie health outcomes [19, 20]. On the other hand, the Andersen and Newman framework gives insights into conditions that either facilitate or impede access and utilization, and are categorized as predisposing, enabling factors, and the perceived need for health care services [21]. The Andersen and Newman framework of health services utilization has been widely used to understand barriers to access and utilisation of HIV prevention and care services among other high-risk populations [22–24].

## Methods

### Study area

This study was conducted in the Greater Kampala metropolitan area (GKMA). The GKMA occupies about 970 km^2^ of land and geographically encompasses a circle of about 30 km radius from Kampala city centre. The GKMA comprises Kampala capital city, Mukono, and Wakiso districts. This is also the most populated region in Uganda. Wakiso district has the highest population in Uganda, followed by Kampala, with a population of approximately 2 million and 1.5 million people, respectively, whereas Mukono district is the 7th most populated in Uganda, with 596,804 people [25].

### Study population

This study was conducted among trans-women sex workers in the GKMA. Trans-women sex workers were defined as women aged 18 and above who were assigned male at birth, self-identified as women sex workers and had received money or goods in exchange for sexual services in the last 1 month.

### Study design, data collection procedure and tools

A formative qualitative study was conducted. Implementation of the study was spearheaded by researchers from the Makerere University School of Public Health in collaboration with the programs department of Transgender Equality Uganda (TEU). TEU played a central role in linking the study team to trans-women sex workers, and all non-governmental organisations working with transgender persons. Besides, some trans-women sex workers participated in data collection and mobilisation of study participants. Interviews were conducted with trans-women sex workers who had sought Sexual and Reproductive Health (SRH) from 7 key population-friendly healthcare facilities (3 public and 4 private) in the GKMA. At the time of data collection, the study participants were purposively selected and recruitted using the snowball tecnique.

We conducted 9 focus group discussions (FGDs), each comprising between 7 to 8 participants, 3 per district using an FGD guide (Additional file 1). The FGDs provided an opportunity to capture views on access and utilization of HIV/STI prevention and care services from the trans-women sex workers community. Specifically, these FGDs were used to explore the SRH issues that affect the trans-women sex workers, their health-seeking behaviours, and attitudes towards available healthcare services as well barriers faced. In order to ensure privacy and confidentiality, FGDs were conducted in secluded places where trans-women sex workers usually gathered during their free time. These included entertainment places like restaurants or hotels. The actual time for the interview was agreed on with the selected study participants. Interviews were conducted during the day after 11.00 am when most trans-women sex workers were awake and before going for work in the evening. With help of an IDI guide (Additional file 3), we conducted a total of 22 follow-on in-depth interviews (IDIs) (10 for Kampala city, 6 for Mukono and 6 for Wakiso district) with selected trans-women sex workers from FGD participants. The IDIs provided an opportunity to capture the lived experiences, and to gain a deeper understanding of SRH issues identified during FGDs. A Key Informant Interview (KII) guide (Additional file 2) was administered to a total of 6 key informants who included policy makers at the Ministry of Health and managers of healthcare facilities (both private, public and key population-friendly healthcare facilities), where the trans-women usually access SRH services. Data were collected on the main SRH problems faced by the trans-women sex workers' community, experiences while accessing HIV and STIs prevention and care services, availability of SRH services, delivery of SRH services, quality and attitudes towards available SRH services, and barriers to utilisation of SRH services. The FGD, KII and the IDI guides were developed and modified by the study team following a thorough review of literature on access to HIV/STI prevention and care services among trans-women sex workers. The total number of IDIs and KIIs conducted was determined by the principle of theoretical saturation [26].

### Data management and analysis

All interviews were digitally recorded with permission from the study participants. These were then transcribed verbatim by two experienced transcribers. Interviews conducted in the local dialect were also transcribed and translated by experienced bilingual personnel without losing meaning. Transcripts were read several times by two members of the study team, who then developed codes and code book definitions based on the study objectives while integrating emerging themes from the data. The code book was discussed and agreed upon by all members of the core study team. Coding of the transcripts was done using ALTAS-ti software in order to ease analysis. The code reports generated by ALTAS-ti were read and discussed by study investigators who afterwards agreed on themes, organising themes and basic codes. A social ecological model was used in the presentation of findings.

### Quality control

Research assistants with a good command of English and the local language (Luganda) were trained to ensure validity and reliability of the study results. The interview guides were back- translated from English to the local language by two experienced translators. The original English version was compared with the version translated from the local languages to ensure consistency of meaning. Furthermore, research assistants were also oriented on the common slangs and terminologies used by the trans-women sex workers' community.

## Results

The median age of study participants for IDIs was 21.0 years. The majority, 90% (20/22) of the participants were aged between 18 and 24 years; 50% (11/22) had attained an ordinary level of education, and 72% (16/22) had never been married. About 50% (11/22) of the study participants did not have any other source of income other than sex work, and 55% (12/22) stayed with a fellow trans-woman. With regard to sex work, 55% (12/22) had engaged in sex work for less than 5 years, and the majority, 41% (9/22) operated from their residences or homes. Less than half of the study participants, 36% (8/22) had ever tested for HIV. (Table 1).

**Table 1:** Characteristics of the study participants for in-depth interviews

Variable	Category	Frequency (***N*** = 22)	Percentage (%)
**Age**	18–24 years	20	90.9
	25–27 years	2	9.1
**Highest level of education**	Primary	1	4.5
	O′ level	11	50.0
	A level	8	36.4
	University	2	9.1
**Marital status**	Divorced/Separated	1	4.5
	Single, never married	16	72.7
	Not Married but in relationship with biologically male born person	4	18.2
	Widowed	1	4.5
**Religion**	Catholic	9	40.9
	Muslim	5	22.7
	No religion	1	4.5
	Pentecostal	2	9.1
	Protestant	5	22.7
**Other sources of income other than sex work**	Casual Worker	3	13.6
	Other Businesses	4	18.2
	Salaried	4	18.2
	No other source of income	11	50.0
**Who do you currently live with?**	Live with fellow trans-woman	12	54.5
	Live with family members	6	27.3
	Live alone (no one to stay with)	4	18.2
**Type of sex work**	Street based	3	13.6
	Entertainment place based	7	31.8
	Residence/home-based	9	41.0
	Online/phone	3	13.6
**Duration in sex work**	Less than 5 years	12	54.5
	More than 5 years	10	45.5
**Ever tested for HIV**	Yes	8	36.4
	Missing information/refused to answer	14	63.6
**Frequency of testing for HIV (*N*** **= 8)**	Three or more times per year	7	87.5
	Twice	1	12.5

Emerging themes from our data were classified as barriers to access and utilisation of HIV/STI prevention and care services, and coping strategies. The “barriers theme” encompassed the following organising themes; individual level barriers, community level barriers and healthcare system barriers. The healthcare system barriers, unless stated otherwise were evident at both the general (private and public) and key population-friendly healthcare facilities. The “coping strategy theme” encompassed the following organising themes; coping with lack of lubricants and STI drugs and coping with stigma and discrimination. The findings are presented following these themes. (Table 2).

**Table 2:** Barriers to access and utilisation of HIV/STI prevention services and coping strategies among trans-women sex workers in the greater Kampala metropolitan region, Uganda

Themes	Organising themes	Basic themes
**Barriers**	Individual level barriers	Internalised stigma
		Low socio-economic status
	Community level barriers	Transphobia
	Healthcare system barriers	Social exclusion and lack of recognition by other key population groups
		Stigmatisation by some healthcare providers at key population-friendly healthcare facilities
		Breach of confidentiality by some healthcare providers
		Limited hours of operation of some key population-friendly healthcare facilities
		Discrimination by some healthcare providers due to the transgender identity
		Discrimination by straight patients because of being a transgender
		Stockout of STI drugs, lubricants and other medical supplies
		Inadequate availability of well-equipped treatment centres
		High cost of drugs
**Coping strategies**	Coping with inadequate access to lubricants and STI drugs	Use of substitutes such as lotions, avocado, egg white or yoghurt to cope with a lack of lubricants
		Resorting to use of herbs to cope with inadequate access to STI drugs
	Coping with stigma and discrimination	Use psychoactive substances to cope with stigma and discrimination
		Changing the dress code to hide their preferred gender identity

### Barriers to access and utilisation of HIV/STI prevention and care services (Table 2)

#### Individual level barriers

##### Internalised stigma

Internalised stigma among trans-women sex workers was mentioned as a key barrier to access and utilisation of HIV/ STI prevention services. According to the study participants, some trans-women felt once they went out to seek healthcare services, they would not be welcomed by healthcare providers, while others felt that the public would accuse and pinpoint at them as those pretending to be what they are not. Internalised stigma among trans-women sex workers was portrayed in the following forms.

**Perception of being viewed as abnormal**

This study revealed that some trans-women sex workers suffer from trauma, mainly resulting from the perception of being viewed by the rest of the community as being abnormal. This feeling hindered trans-women sex workers from associating with other members of the community, consequently preventing them from accessing information on SRH services.

*“When the community around gets to know that you are a transgender, you feel shy to move out of the house; you cannot be bold enough. Whenever you are sick, there is no one you can talk to because of trauma. Now like for my situation, I understand that there are other people who may think that am abnormal and they do not like it. So, I think that is another factor that hinders me from going to those health centres” (IDI participant).*

**Low self-esteem**

Trans-women sex workers found it difficult to identify with their gender, sex work and health condition while at healthcare facilities, thereby making diagnosis of SRH conditions difficult. It was also evident that some trans-women sex workers were afraid of mentioning that they had sexual feelings for men and associating themselves with sex work.

*“It is difficult to express ourselves while at healthcare facilities. I cannot easily say that I am a trans-woman or even a sex-worker, and that I am suffering from this condition (STI) or that, yet they do not understand who we are. Again, having a low self-esteem because the trans-women are not well recognized as a key population.” (IDI participant).*

*“Explaining to somebody that you are a trans-woman is not easy. Telling a healthcare provider that “I am a trans-woman, I behave like a woman although I am a man, my sexual feelings are for men, I am only attracted to men, so, I have sex with men and I am actually a sex-worker.” If you say it today, you will also need to explain to another healthcare provider next month. It is not easy!” (IDI participant).*

**Feeling embarrassed to declare STIs**

Trans-women sex workers reported to have feared healthcare providers, felt embarrassed or did not have the confidence to report the STIs they suffered from, while at both general and key population-friendly healthcare facilities, thereby affecting access to SRH services.

*“Most Trans-women lack the confidence to express themselves, and fear healthcare providers. If I know that I have STIs like anal warts, I feel embarrassed to tell the healthcare provider what I am suffering from. Therefore, as the trans, we need that confidence to be able to express ourselves and what we are suffering from to the healthcare providers in order to get the treatment we need.” (IDI participant).*

Internalised stigma among trans-women sex workers was also highlighted by one of the key informants who mentioned that:

*“Trans-women sex workers also stigmatize themselves. They often think that they will be discriminated against while at healthcare facilities. They also at times believe that they will be referred to as homosexuals yet they are not” (Key informant).*

#### Low socioeconomic status

It was evident that many trans-women sex workers have a low socioeconomic status and rely solely on sex work as their source of livelihood. A limited source of income implied that many trans-women sex workers failed to access SRH services at both the general and key population-friendly healthcare facilities due to lack of money to pay for transport and to foot medical bills.

*“The first issue is the distance from the healthcare facility. I for example, stay in Buziga [suburb] and the healthcare facility is in Namuwongo [another suburb]. You may think that it is a short distance, but once you’re “broke” [lack of money] you can actually fail to access it. You may also get an STI and fail to get treatment due to lack of money” (FGD).*

*“Poverty is one of the challenges that hinder trans-women sex workers from accessing HIV/STI prevention and care services. Some trans-women sex workers claim that they do not have transport to come and access SRH services. So, they would rather sit back and not access the services” (Key informant).*

#### Community level barriers

##### Transphobia

Some trans-women sex workers were not able to access both general and key population-friendly healthcare facilities due to fear of community perpetrated violence and gossip. Trans-women sex workers were discriminated against by the community because they defied societal expectations such as dress code and behaviour. As a result, many trans-women sex workers tend to socially exclude themselves and only move out of their homes to visit bars at night.

*“Some trans-women sex workers fear that they may be attacked by the community because of the way they dress, and of course the society expectation. If you are a man, you are supposed to dress and walk like a man, yet, you know the trans-women dress as females” (Key informant).*

*“We fear community gossip and sometimes physical assault. Actually, due to fear and community gossip, most trans-women sex workers travel at night to visit bars. It is rare to find us visiting friends during the day because even gay men do not welcome us. My husband can’t allow any unknown person to enter our house, they are trans-phobic” (IDI).*

#### Health system barriers

##### Social exclusion and lack of recognition at some key population-friendly healthcare facilities

Trans-women sex workers were branded by healthcare providers at some key population-friendly healthcare facilities as “proud” because they did not want to associate, and had a poor relationship with other members of the key population group, particularly the lesbians, gays and female sex workers. The poor relationship with other members of the Lesbians, Gay, Bi-sexual, Transgender and Intersex (LGBTI) community mainly stemmed from competition for sex clients and wanting preferential treatment while at healthcare facilities. Wanting special treatment was irrespective of whether it was a general, key population-friendly or an NGO-managed healthcare facility. Failure to get the preferential treatment at the different healthcare facilities stopped some of them from seeking HIV and other STI prevention services.

*“They (trans-women sex workers) do not want to associate with the rest of the LGBTI community. They always want special treatment yet the LGBTI community here does not recognize them”. (Key informant key population friendly healthcare facility).*

*“Trans-women sex workers always want to be treated in a very special way when they come to the healthcare facility. They always want to be served faster than other key populations. They are proud, very proud, and always want to be handled like princesses. They think they are better than the lesbians, and often despise other genders. That is why, in most cases, they are not always welcomed by other members of the LGBTI community. We always find that challenge. If they find a queue of other patients, for instance patients who may have come in the morning, they always want to bypass and enter the clinical room and tell you “handle me and I go”. When you tell them to wait in the queue they reply; “Musawo (healthcare provider), I have told you I want to go, I have to go and sell sex” so they see the other genders as competitors for sex clients so they always want to be served first”. (Key informant NGO managed healthcare facility).*

##### Stigmatisation by some healthcare providers at key population-friendly healthcare facilities

Some trans-women sex workers were often embarrassed and stigmatised by healthcare providers who saw them as a disgrace and people pretending to be what they are not. Some healthcare providers even went to an extent of telling them to quit “homosexuality”, threatening that they (trans-women) would even miss heaven.

*One of us had gone for a check-up at healthcare facility XY (key population healthcare facility) because the client she had had sex with the previous night was not feeling well. When she entered the doctor’s room, the doctor started preaching to her while saying “why don’t you stop practicing homosexuality? You are going to miss heaven.” (FGD).*

##### Breach of confidentiality by some healthcare providers

Trans-women sex workers reported that some healthcare providers in both general and key population-friendly healthcare facilities breached the ethics principle of confidentiality. As a result, some healthcare providers disclosed information about their patients' (trans-women sex workers) gender identity to other healthcare providers, and other patients. This was seen as a deterrent to seeking healthcare services from these healthcare facilities.

*“Ohhh my God, it’s really a challenge (breach of confidentiality). Sometimes, they (healthcare providers) back bite us, they can go and tell other health workers or patients what has happened while in their office. Some healthcare providers are too religious, some are too much into their culture. They think a man is supposed to be a man and a woman is supposed to be a woman, period! That’s what they perceive. So, by the time you get out, all healthcare providers and patients are aware of who you are.” (IDI).*

##### Limited hours of operation in key population-friendly healthcare facilities

Access and utilisation of HIV prevention and care services in key population-friendly healthcare facilities was hindered by the limited hours of operation of key population-friendly healthcare facilities. Majority of these healthcare facilities did not operate 24 h and were also closed over the weekends. This meant a discontinuity in access to services when needed.

*“Facility XXX closes at 4:00 pm, after which you cannot access any service, yet they don’t work over the weekend. So, it’s really a challenge. They also don’t work at night so if you get any problem at night, you will not be worked on. There are times when these healthcare facilities (key population-friendly healthcare facilities) are closed for example over the weekends, which prevents you from getting the treatment on time.” (FGD).*

##### Ignorance of healthcare providers about the transgender identity

It was common in both general and key population-friendly healthcare facilities to find healthcare providers that were ignorant about the transgender. Respondents revealed that some healthcare providers, despite working in key population-friendly healthcare facilities did not understand the transgender identity. In reference to a key population-friendly healthcare facility, one of the trans-women was quoted;

*“I am going to talk about XXX, that is where I get my treatment. Before I came to Kampala, I was staying in Mukono. One day, I was arrested and beaten severely while in Mukono. So, my friend took me to that healthcare facility (Key population-friendly healthcare facility) for treatment. I had put on a trouser and a shirt but my hair was woven. When I arrived at the health facility, the health worker asked me; “who are you? What are you? I don’t understand you!” I explained to her that I am a trans-woman. She asked me, “who are the trans-women? I don’t understand the trans-women.” The health worker did not have any idea about the trans-woman. She did not have any information about the trans-women. The health worker came and attended to me but she said; “But I have not understood you.” After three days, I went back but I did not meet the one I had explained myself to that I am a trans-woman. That day I found a different one, and this time it was worse. I was very sick and had to be admitted. When I was on the bed, the health provider asked me, ‘what are you? I don’t understand you. I expected you to have breasts because you have plaited your hair. I don’t understand your things.”*

Ignorance of the healthcare providers on the transgender identity was also evident in public and private general healthcare facilities. From the quote below, it was evident that some healthcare providers do not know the difference between the trans-women and the MSMs.

*“I will talk about my experience at YYY facility., It is my friend who took me to this healthcare facility. The first time I went to the healthcare facility, healthcare providers asked me a number of questions. They asked me why I behave like a woman and I told them that I am a trans-woman and that is why I behave like a woman. Again, they asked; “What are you? Are you gay?” They had not been taught much about us.”*

##### Discrimination while at healthcare facilities

Besides internalised stigma, discrimination by the communities where they live and members of other key population groups such as female sex workers and men who have sex with men, the trans-women sex workers reported being harshly judged by some healthcare providers in both the general and some key population-friendly healthcare facilities. Trans-women sex workers also felt that they were not satisfactorily recognized under the key population umbrella by other key population groups and healthcare providers, despite being the face of the LGBTIQ community. It was due to this discrimination and lack of recognition that many trans-women sex workers shunned visiting healthcare facilities for SRH services. The forms of discrimination are elaborated in the following subthemes.

##### Discrimination by some healthcare providers

It was evident that some trans-women sex workers were discriminated by healthcare providers in general and key population-friendly healthcare facilities on the basis of their gender identity. Some respondents pointed out that they were made to wait for longer hours as healthcare providers in both general and some key population-friendly healthcare facilities called upon their colleagues to come and see an ‘abomination’ (a trans-woman) at the healthcare facility. It was also mentioned that some healthcare providers, particularly in general healthcare facilities shunned providing SRH services to some trans-women sex workers, a problem that the respondents attributed to their dress code and physical appearance, which was not considered by some healthcare providers as being normal. In response to the main challenges that trans-women sex workers faced while at general and some key population-friendly healthcare facilities, some respondents were quoted;

*“Some healthcare providers make us appear like tourist attractions. When you get to a healthcare facility, she (healthcare provider) first calls her colleagues saying; “you come and see this abomination (while referring to a trans-woman)”. They make you sit and wait for drugs so that her colleagues can come and look at your appearance. Sometimes you feel out of place (embarrassed) and just decide to leave the healthcare facility without getting the SRH service you want”. (FGD).*

*“Some healthcare providers do not want to treat us because we are trans. I may do my makeup and carry a hand bag as I go to the facility. But once you reach the healthcare facility, you may hear her (healthcare provider) call a colleague; “you come and work on this one, am very busy”. Some of them just don’t want to work on us”. (FGD).*

##### Discrimination by straight patients in public and private general healthcare facilities

Trans-women sex workers pointed out that discrimination by straight patients in general healthcare facilities was prevalent, and prevented them from accessing and utilising SRH services. These mentioned that there was a common practice of straight patients back biting them due to their gender identity. As a result, some are forced to leave the healthcare facility even before interacting with the healthcare provider.

*“It is worse with fellow patients. Once you meet them, it is like you have met fire. They see you as Satan! They judge us as very evil”. (FGD).*

*“I was dressed up in my clothes but not sexy. So, when I reached the healthcare facility, other patients started pointing at me while telling their friends, “come and see the homosexuals”, a man who takes himself to be a woman.” (FGD).*

*“Imagine going to seek health services in the general healthcare facilities and your fellow patients start back biting you. They say; “Look at this one, now what has brought him here.” Then they make fun of you, they laugh at you. So, there is a way even as you are waiting in the general clinic, you are being harassed. So, if you cannot not be patient you may even leave before you see the doctor because of fellow patients.” (IDI).*

##### Stockout of hormones, lubricants, STI drugs and other medical supplies

The utilisation of a wide range of HIV and other STI prevention and care services at key population-friendly healthcare facilities was hindered by stock out of STI drugs, lubricants and hormones (oestrogen-based pills). Availability of lubricants at some key population-friendly healthcare facilities attracts some trans-women to also seek other HIV or STI prevention and care services and vice versa. However, respondents reported that there was inadequate access to hormones required for expression of feminine characteristics. The few who are able to access hormones bought them from private pharmacies with neither prescriptions nor counselling on how to use them from trained personnel. The lack and improper use of hormones dampened the desire of some trans-women sex workers to undergo transitioning. Besides, stockout of STI drugs, lubricants and other medical supplies at key population-friendly healthcare facilities was attributed to the limited supply of such items from the central medical stores. Additionally, some healthcare facilities failed to conduct STI tests due to limited funding of SRH services.

*“There are times you go to a healthcare facility and you don’t find medicine, yet, it is the only place you’re comfortable with. There is a healthcare facility where I get my medication, they know me very well. They usually welcome me, hug me and I feel at home while with healthcare providers. However, there are times I go to the healthcare facility and they tell me that the drugs are out of stock, and this can take like three to four months”. (IDI).*

*“We have challenges of drug stock outs. Healthcare facilities may not be able to offer some of the tests if there are no kits. Right now, healthcare facility XXX does not have enough funding and is not able to do STI tests”. (Key informant).*

Managers of healthcare facilities and ministry of health officials re-emphasized that stock out of STI drugs and other medical supplies was a challenge to most of the healthcare facilities in Uganda. On being asked the biggest challenges faced by healthcare facilities in the delivery of HIV and other STI prevention services, one of the healthcare facility managers of an NGO-managed key population clinic responded;

*“Drugs! Some trans-women want to transition but we have a challenge of hormones. Many people want to start using hormones but we do not have them. So, if they have money, they will run to Nairobi, Kenya or other private pharmacies” (Key informant).*

Healthcare managers acknowledged that drug stockouts compromised the treatment of STIs among the trans-women and the general population at large. Whereas it was revealed that STI testing was done every three months, treatment remained a challenge. However, it was pointed out that strides had been made by some stakeholders to equip key population-friendly healthcare facilities with essential STI drugs.

*“STI treatment has been “hmm … ” a challenge, but it has been routinely offered whenever HIV testing is being done. We have been able to screen for STIs … as we do HIV tests. Therefore, screening is being done, but the problem is treatment for those who are found with STIs. Most public healthcare facilities do not have enough STI drugs. However, efforts are being made by PEPFAR to stock key population healthcare facilities with STI drugs. Although we still hear that some facilities have stockouts for now, yes.” (Key informant).*

Drug stock outs were further validated through a key informant interview with one of the Ministry of Health officials who pointed out that they had obtained similar evidence in a recent validation study for STI and Prevention of Mother to Child Transmission (PMTCT). Furthermore, it was found out that most of the STI drugs in supply were not liked by patients, and that, STIs regimens being supplied by the central medical stores to healthcare facilities were not those recommended by the new STI treatment guidelines. As a result, trans-women sex workers experienced a recurrence of STIs.

*“One of the major problems is limited supply of drugs. We recently conducted a validation study for treatment of STI and PMTCT and also discovered that STI drugs are not liked by patients, and are expensive.” (Key informant).*

*“The National Medical Stores doesn’t supply the drugs that were recommended by the 2010 STI treatment guidelines. Even when you are in meetings with the health workers, they tell you that “you are telling us to treat STIs using drugs that are not being supplied”. And, if you are not supplying them, they resort to treat STIs using drugs recommended in the old treatment guidelines, of which research was done and most of those treatments are not helpful to the patient.” (Key informant).*

##### Inadequate availability of well-equipped treatment centres

Trans-women sex workers faced challenges in accessing STI drugs from general healthcare facilities. This was attributed to the fact that most general healthcare facilities serve heterosexual populations which may not suffer from STI in peculiar parts of the body such as anal region. Such infections require proctoscopy for better diagnosis and management, yet, it is not readily available in the general healthcare facilities.

*“There are times you go to a healthcare facility and the equipment are not available. The infections we suffer from are different from those of straight people, especially infections that are sexually transmitted. You cannot go to general healthcare facilities to seek such treatment because they do not have those specific machines. So, you have to go to key population-friendly healthcare facilities like XXX and YYY for such treatment. You cannot just go to other healthcare facilities because even talking to the healthcare provider will be a problem. You cannot easily open up and tell them, I have this infection on the anal area. They will not understand you.” (IDI).*

##### High cost of STI drugs

Respondents pointed out that, besides unavailability, they found it expensive to buy STI drugs from general healthcare facilities. Some argued that they only depended on sex work thus making it difficult for them to save money to drugs.

*“The problem we always get is that when you get to the healthcare facility and ask for treatment, they tell you that some of the medicines are not in stock, which means you have to use some of the money the client paid you to go and buy the medicine. In most cases, the medicines are very expensive. So, you end up spending all the money in buying medicine and saving nothing”. (FGD).*

*“Sometimes our clinics (key population-friendly healthcare facilities) do not have the medicines yet we may be in need of them. You may also find that you do not have money to buy from the pharmacy since these are very expensive. For instance, the cost of treatment for anal warts ranges from UGX 25,000 (equivalent to 7 USD) to UGX 40,000 (equivalent to 11 USD) which I don’t have since I mainly depend on sex work” (IDI).*

### Coping strategies (Table 2)

#### Use of substitutes such as lotions, avocado, egg white or yoghurt

Some trans-women sex workers used lotions while others made local lubricants by blending avocado, egg white, margarine, or yoghurt to cope with lack of lubricants. However, the use of lubricants made of yoghurt, margarine and jellies was associated with side effects such as a burning sensation in the anal area, abdominal pain and diarrhoea. When asked how the trans-women sex workers coped with a lack of lubricants, some of the IDI participants said;

*“Improvising! It’s actually a funny story. We use eggs! You get an egg; remove the yolk and you remain with albumen. It’s what we use as lubricants. That’s option one. Option two; We use yoghurt; the PH of yoghurt is not really harsh and aggressive to the rectum. If not, yoghurt and avocado, you get the avocado, blend it to get a smooth cream because avocado has an oily part. And the last option would be ‘blue band (brand name for margarine)’ or margarine” (IDI).*

*“At times we go to meet clients without lubricants due to lack of money to purchase them. Therefore, we decide to use quick items like oils or jelly to lubricate “down” (anal area). These items have chemicals that can even burn the skin. As a result, the anus changes and turns red, and it feels like it is burnt. Some of these oils reach inside the digestive system and changes the internal system, you start feeling stomach ache, and diarrhoea. You may also realize that the behind is full of wounds to the extent that you can’t do anything by yourself.” (IDI).*

#### Psychoactive substances use

Trans-women sex workers resorted to the use of psychoactive substances such as alcohol in order to cope with discrimination, reduce stigma and depression, and to increase their esteem. Some trans-women sex workers mentioned that the use of psychoactive substances would boost their confidence thereby making it easy to approach healthcare providers for SRH services. When asked how they coped with stigma, one of the IDI participants said;

*“As transgender persons, we sometimes use drugs to gain confidence, fight off depression or to relieve ourselves of stigma” (FGD).*

This was also reaffirmed by one of the healthcare providers who said;

*“Drinking alcohol and the use of drugs; anyway, when I ask them why they drink or use drugs, they say the reason is to cope or survive.” (Key informant).*

### Use of herbs to treat STIs

Some trans-women sex workers resorted to the use of herbal medicines to treat STIs such as syphilis in order to cope with a high cost, limited access and stockout of STI drugs.

*“We have these herbalists who claim to cure STIs, and some of us have used these herbal medicines to cure these infections. Therefore, they (herbalists) are alternatives. Some trans-women sex workers go to these “Sengas” (female herbalists) and take natural herbs or botanical plants that have healing properties for STIs like syphilis.” (IDI).*

#### Changing the dress code to hide their preferred gender identity

Trans-women sex workers in this study pointed out that they often dressed in a way that met the societal expectations. The trans-women pointed out that prior to accessing any general or key population-friendly healthcare facility they had to assess the environment and then dress accordingly. The respondents revealed that it was difficult for them to cross-dress, even on their way to a key population-friendly healthcare facility since these had to traverse the community before reaching the healthcare facility. Consequently, these dressed in “men’s trousers” as opposed to their desired feminine attires.

*“What really happens when we want to go to any healthcare facility is that we look at our environment. Cross dressing would bring me a lot of problems, and if am to walk or access a healthcare facility, I have to wear like a normal man so that I don’t attract people’s attention.” (IDI).*

*“If I am to access a facility, I dress like a normal man, without a jegging or even tight pants. I just behave like a normal person. That is the only way you can access a healthcare facility without being discriminated.” (IDI).*

## Discussion

This qualitative study explored barriers to access and utilisation of HIV/STI prevention and care services among trans-women sex workers. The themes that emerged from this study were classified as individual, community and healthcare system barriers to access and utilisation of HIV/STI prevention and care services. It is worth noting that this specific subgroup faces both access and utilisation challenges related to gender identity and transphobia, as well as those related to sex work. In this section, we discuss the implications of existing barriers to the future of prevention and treatment of HIV and other sexually transmitted infections in low resource and transphobic settings. Furthermore, we endeavour to elaborate how trans-women sex workers cope with the existing barriers, in a setting that is unquestionably transphobic [27].

Background characteristics of our study participants for IDIs indicated that only 8 out of the 22 respondents reported ever testing for HIV. This may not be surprising, given the limited innovations in increasing access to HIV prevention services to this unique key population subgroup. Trans-women sex workers are known to be a socially excluded population [14], which requires innovative approaches in increasing access to SRH services such as HIV testing and treatment of STIs. This would also require breaking the structural barriers at individual, community and healthcare facility level, thus creating an enabling environment for access and utilisation of existing services.

At individual level, internalised stigma, expressed through a low self-esteem, perception of being viewed as abnormal, feeling embarrassed to identify with the sex work, and feeling embarrassed to declare STIs not only compromises prevention efforts but also the quality of care obtained at both general and key population-friendly healthcare facilities. Trans-women sex workers in our study feared to declare STIs, especially to private healthcare providers and those working in public healthcare facilities without specialised key population-friendly healthcare facilities. This is so because trans-women sex workers suffer infections in unique parts of the body such anal warts, which would arouse curiosity from healthcare providers to find out the underlying causes. This could in turn force transgender persons to declare their gender identity and occupation as sex workers, which they may not want due to fear of discrimination, stigmatisation and legal consequences. Our findings reaffirm those by Ganju and Saggurti [28] and Rael, Martinez [29] which indicated that internalised stigma, including a low self-esteem affected access and utilisation of HIV prevention and care services among transgender persons. In addition, Fisher, Fried [30] and Chambers, Rueda [31] pointed out that transgender persons may not discuss their sexual healthcare needs with healthcare providers because they anticipate being stigmatised, and a breach of privacy and confidentiality by healthcare providers. Based on this school of thought, it is not surprising that less than half of our study participants had ever tested for HIV. It is also worth noting that some trans-women sex workers resorted to the use of psychoactive substances such as alcohol and thus increasing their risk to HIV and other STIs.

A low socio-economic status, evidenced by half of the study participants not having an alternative source of income hindered access and utilisation of HIV/STI prevention and care services. The low socio-economic status of trans-women sex workers could be due to the fact that they are denied education and employment opportunities [32]. This can limit their ability to reach healthcare facilities. It may not be surprising that trans-women sex workers in close proximity to public healthcare facilities may not bother to attend them due to the fear that they will be requested to buy drugs for STIs, after all most healthcare facilities acknowledged persistent drug stockouts. This coupled with a high cost of STI drugs, forced many trans-women sex workers to resort to the use of traditional medicines such as herbs for the treatment of STIs such as syphilis. The use of herbal treatments for treatment of STIs among transgender persons in Uganda has also been reported by [33].

Transphobia including violence greatly compromised decisions to seek appropriate medical help, reaching an appropriate healthcare facility and receiving adequate care. The fear of violence, expressed in forms of physical assaults and gossip not only affects the social life but also health seeking behaviours of transgender persons. Issues of violence toward transgender persons in Uganda are widely documented [34–38]. Due to fear of violence, trans-women sex workers in our study coped by changing the dress code to the one that meets societal expectations in order to access HIV/STI prevention and care services, despite not being in conformity with their gender identity. The use of dress code to mask the gender identity of transgender persons has been reported in Malaysia and the United States [39, 40].

The current study reveals that trans-women sex workers did not access services due to the lack of recognition by other key population groups such as female sex workers and MSMs at some key population-friendly healthcare facilities. The lack of recognition by other members of the key population umbrella forced some trans-women into social exclusion. The lack of recognition and social exclusion that characterises the trans-women in our study could be due to the fact that trans-women sex workers are seen as competitors for sexual partners and clients. Besides being competitors for sexual clients and partners, lack of recognition and discrimination by other key population groups might have arisen due transgender behavioural dynamics, gender identity and physical appearance [41].

Our study brings to light stigmatisation of some trans-women sex workers by some healthcare providers at key population-friendly healthcare facilities as hindrance to access and utilisation of HIV/STI prevention and care services. Whereas key population-friendly healthcare facilities are known to be receptive to their target group, it was not always the case in our study setting. This may be attributed to the fact that many key population programs in Uganda focus on the female sex workers, lesbians and MSMs, largely ignoring the transgender. Besides, the knowledge gap on trans-women sex workers in Africa limits funding to trans-specific HIV/STI prevention, treatment and care interventions [42], including those targeting a reduction in stigmatisation and psychosocial support.

Breach of confidentiality by some healthcare providers in both general and key population-friendly healthcare facilities hindered some trans-women sex workers from accessing and utilising HIV/STI prevention and care services. The breach of confidentiality could be as a result of transphobia and the trans-women engagement in sex work. Besides, a few healthcare providers have been trained on the transgender identity and their involvement in sex work to enable them change their perception towards this specific sub group. Moreover, our study reports that healthcare providers in general and some in key population-friendly healthcare facilities were ignorant about the transgender identity and some viewed the trans-women as “tourist attractions.” The breach of confidentiality of the trans-women, including those engaged in sex work in healthcare settings has recently been documented [43]. Therefore, in order to achieve better HIV/STI prevention and care outcomes for trans-women sex workers, there is need to train all healthcare providers including in the use of chosen names and pronouns and how to avoid breaches of confidentiality.

This study revealed limited hours of operation in key population-friendly healthcare facilities as a hindrance to the access and utilisation of HIV/STI prevention, care and treatment services. The fact that majority of the key population-friendly healthcare facilities in the greater Kampala Metropolitan area operate during weekdays and from 8:00 am to 5:00 pm, limits the trans-women sex workers access to HIV/STI prevention and care services especially during the weekends and at night when services are most needed. Access to services like pre- and post-exposure prophylaxis is required irrespective of time of the day. Limited hours of operation as a barrier to utilisation of HIV/STI prevention services in Uganda was also reported by Magala, Tapati [44]. A review of the hours of operation of key population-friendly healthcare facilities would therefore, be critical in improving access and utilisation of HIV/STI prevention and care services. Besides, key population-friendly healthcare facilities may explore the use of peer leaders and other community health workers in improving access to HIV/STI prevention services, beyond the usual hours of operation of healthcare facilities.

This study revealed that discrimination of the trans-women by some healthcare providers in both general and key population-friendly healthcare facilities, and straight patients barred them from accessing HIV/STI prevention and care services. The discrimination reported in our study could be due to the fact that trans-women sex workers do not behaviourally conform to the expected gender norms and roles, and thus prone to transphobia from healthcare providers and straight patients. To put this into context, some trans-women sex workers visit healthcare facilities dressed in a way that is against the expectation of healthcare providers and other patients in these healthcare facilities. Therefore, it is important to break all forms of discrimination by healthcare providers and other patients in order to improve disclosure and confidence in the healthcare system, and consequently access to quality sexual and reproductive healthcare services. Our findings are not different from those of Ganju and Saggurti [28], Poteat, Reisner [32] and Bradford, Reisner [45] which indicated that trans-women sex workers face widespread discrimination in healthcare. Additionally, this study re-echoes the importance of non-discriminatory and inclusive SRH services for trans-women sex workers [46].

The current study also revealed that a stockout of hormones, lubricants and an inadequate access to STI drugs was pronounced in both general and key population-friendly healthcare facilities. Availability of hormones and lubricants often facilitates access and utilisation of other HIV/STI prevention and care services. However, unavailability of lubricants deterred some trans-women sex workers from visiting healthcare facilities, and consequently accessing HIV/STI prevention and care services. Trans-women sex workers coped with inadequate access to lubricants by using substitutes such as lotions, margarine, avocado, egg white or yoghurt. The use of eggs, lotions, margarine as a lubricant among transgender persons is documented [38, 47]. However, these may have health effects to the users. It was also reported that some trans-women sex workers resorted to the use of traditional medicines such as herbs for the treatment of STIs. The use of herbal medicines for treatment of STIs including HIV is not novel in Uganda and is widely documented [48–50]. However, there is need to understand the efficacy and health effects of using herbs in the treatment of STIs. The inadequate access to STI drugs and drug stockouts reported in our study have also been found in high-income countries, where it was attributed to failure of drug authorities to provide an adequate supply of medical supplies to healthcare facilities working with transgender persons [51]. In Uganda’s context, however, a limited supply of essential drugs and lubricants could also be associated with limited funding for SRH interventions, and transphobia not only among healthcare managers but also policy makers. Due to inadequate access to STI drugs, healthcare managers and policy makers pointed out that healthcare providers had resorted to the use of regimens that were not being recommended by the current STI treatment guidelines. This could exacerbate antimicrobial resistance thereby making the treatment of these conditions more expensive.

### Strengths and limitations

This is one of the few studies that has so far examined barriers to access and utilisation of HIV/STI prevention and care services among trans-women sex workers in a low resource setting. However, our participants for FGDs were not a homogenous group as per socio- demographic characteristics in terms of age group, education level, tribe, and religious background. Our intention was to capture the commonalities of lived experiences of trans-women sex workers who live in Greater Kampala and not differences in socio- demographics. In addition, findings from FGDs were triangulated with findings from IDIs and key informants, thus increasing credibility of findings. According to the Ugandan culture a topic on sex is considered very sensitive issue. Besides sex work is illegal in Uganda. This could have led some participants not to fully share some lived experiences, although there was trust build between our research assistants and study participants. For this study we only interviewed trans-women sex workers from the Greater Kampala metropolitan area, which is an urban setting. Therefore, our results cannot be generalised to all transgender individuals as some of the observed barriers are specifically related to sex work.

## Conclusions

This study revealed that trans-women sex workers face challenges with access to and utilisation of HIV/STI prevention care and treatment services. At individual level, access to and utilisation of HIV/STI prevention and care services are hindered by self-enacted stigma and a low socio-economic status. At community level, the main barrier to access and utilisation of HIV/STI prevention and care services is transphobia, while at the healthcare system level, barriers include: social exclusion and lack of recognition by other key population groups, stigmatisation by some healthcare providers at key population-friendly healthcare facilities, breach of confidentiality by some healthcare providers, limited hours of operation of some key population-friendly healthcare facilities, discrimination by straight patients and healthcare providers at both general and key population-friendly healthcare facilities due to the transgender identity, stockout of STI drugs, lubricants and other medical supplies, inadequate access to well-equipped treatment centres, and high cost of drugs. These findings suggest the need for the different stakeholders to break these barriers through training healthcare providers on trans-friendly services, sensitisation of the community on who the transgender persons are, and increased funding of SRH programs.

## Supplementary Information

**Additional file 1.** Focus Group guide for understanding barriers to access and utilisation of HIV/STI prevention and care services among trans-women sex workers.

**Additional file 2.** Key Informant Interview guide for understanding barriers to access and utilisation of HIV/STI prevention and care services among trans-women sex workers.

**Additional file 3.** In-depth interview guide for understanding barriers to access and utilisation of HIV/STI prevention and care services among trans-women sex workers.

## Data Availability

The data analysed during the current study are not publicly available due to illegality of sex work, hence the need to protect individual privacy and keep the names of healthcare facilities and other socialising places anonymous. However, data are available from the corresponding author on reasonable request.

## Abbreviations

AIDS: Acquired immunodeficiency syndrome
HIV: Human immunodeficiency virus
SRH: Sexual and Reproductive health
STI: Sexually transmitted infections
