# Supplementary material for: Barriers to access and utilisation of HIV/STIs prevention and care services among trans-women sex workers in the greater Kampala metropolitan area, Uganda
Source: BMC Infect Dis. 2020 Dec 7;20:932. doi: 10.1186/s12879-020-05649-5 (PMC7720523; doi:10.1186/s12879-020-05649-5)
Supplement: Supplementary file 1 — Additional file 1. Focus Group guide for understanding barriers to access and utilisation of HIV/STI prevention and care services among trans-women sex workers. [file 12879_2020_5649_MOESM1_ESM.docx]

## Additional file 1

**Focus Group guide for understanding barriers to access and utilisation of HIV/STI prevention and care services among trans-women sex workers**

**DOC file**

FGD guide

Transgender SW Sexual Reproductive Health (SRH) problems and needs

1. What are the main health problems affecting you or other transgender SW like you living in this area? *(probe for SRH problems)*
2. What are the main reproductive health needs of Transgender ***SW*** like you in this community*?*

Seeking HIV and other STIs prevention and care services

1. Now let us focus on HIV and STIs prevention and care services. If one needs HIV and other STIs prevention and care services, where does one get the services/help? *(Probe for all providers including public Private health providers and health facilities. Which of these providers/facilities are most accessible and why?)*

Attitudes of transgender HW towards the available SRH

1. What HIV and STIs prevention and care services are available in the health centres around you? (probe for all RH services such as counselling for HIV and other STIs, Treatment, and availability of condoms).
2. Do you and other young people like you visit the health facilities to obtain HIV and STIs prevention and care services? IF yes, what do you think of these services? *(probe for opinions on quality, accessibility, privacy, care from staff, etc)*
3. Kindly share with us your personal experience while accessing HIV and STIs prevention and care services for the mentioned facilities.

Facilitators to access and utilisation of HIV and other STIs prevention, treatment and care services

1. What are some of the conditions that make easy for you to access and utilisation of HIV and other STIs prevention, treatment and care services

Barriers to access and utilisation of HIV and other STIs prevention, treatment and care services

1. What are some of the conditions that make it difficult for you to access and utilisation of HIV and other STIs prevention, treatment and care services

Suggestions to improve service delivery.

1. What services would you like to be provided for you to help you with your HIV and STIs needs
2. How would you like these services to be provided for you to benefit better? *(probe for location, personnel, cost, service setting)*

Attitudes towards available services

1. In your view, what are the attitudes of transgender SW towards the available sexual and reproductive health services in greater Kampala?
2. What do you think of the quality of services provided to youth in this district/facility?
3. If not sufficient, how should services be improved?
