# Supplementary material for: Barriers to access and utilisation of HIV/STIs prevention and care services among trans-women sex workers in the greater Kampala metropolitan area, Uganda
Source: BMC Infect Dis. 2020 Dec 7;20:932. doi: 10.1186/s12879-020-05649-5 (PMC7720523; doi:10.1186/s12879-020-05649-5)
Supplement: Supplementary file 2 — Additional file 2. Key Informant Interview guide for understanding barriers to access and utilisation of HIV/STI prevention and care services among trans-women sex workers. [file 12879_2020_5649_MOESM2_ESM.docx]

## Additional file 2

**Key Informant Interview guide for understanding barriers to access and utilisation of HIV/STI prevention and care services among trans-women sex workers**

**DOC file**

**KII guide**

Transgender SW Sexual Reproductive Health (SRH) problems and needs

1. In your opinion, what are the main sexual and reproductive health problems affecting most transgender in Greater Kampala Metropolitan regions?

*Evaluation of health facilities (public and private) to* provide HIV and other STIs prevention, treatment and care services

1. Name facilities that provide SRH services including HIV and other STIs to transgender SWs in greater Kampala that you know?
2. How ready would you say these facility/facilities/ are to provide HIV and other STIs services? (check for availability of suitable space/rooms, trained personnel, availability of equipment and medical supplies etc)
3. How many technical staff do you have? Of the staff you have, how many are trained in providing services to transgender SWs?
4. In terms of infrastructure, would you say that they are suited to offer HIV and other STIs prevention, treatment and care services to transgender SWs? If yes how? If no, what do you think is needed?
5. What kind of HIV and STI prevention and treatment services are provided for transgender SWs in this facility (facilities in greater Kampala)?
6. Would you say in your opinion that the available SRH services in your facility are friendly to transgender SWs? Give reasons for your answer

Attitudes towards the available services

1. In your view, what are the attitudes of transgender SWs towards the available sexual and reproductive health services in GKMA? And why
2. What is the quality of health services offered to the transgender SWs in this district/facility? If not sufficient, how should they be improved?

Feasibility of conducting an intervention for improving services for transgender SWs.

1. In your view, what other specific SRH services besides HIV an STIs should be prioritised for transgender SWs in Uganda?
2. In your opinion, how best should the SRH services be provided for maximum benefit of transgender SWs? *(probe for location, personnel, cost, service setting)*
3. If you were to set up an intervention for transgender, what would you offer? (Probe the range of services). Would they or some of them be offered at a fee?
4. How would you package the a) standalone clinics b) integrated services?
5. What strategies would you adopt to ensure that there is integration with existing services
6. If we are to carry out an intervention to improve the provision of services to transgender SWs, how should this be done?
